# Monitoring cardiopulmonary resuscitation quality in emergency departments: a national survey in China on current knowledge, attitudes, and practices

**DOI:** 10.1186/s12873-022-00590-z

**Published:** 2022-02-28

**Authors:** Kang Zheng, Lanfang Du, Yu Cao, Zhendong Niu, Zhenju Song, Zhi Liu, Xiaowei Liu, Xudong Xiang, Qidi Zhou, Hui Xiong, Fengying Chen, Guoqiang Zhang, Qingbian Ma

**Affiliations:** 1 Department of Emergency Medicine, Peking University Third Hospital, Beijing, 100191 China; 2 Department of Emergency Medicine, Sichuan University West China Hospital, Chengdu, 610041 China; 3 Department of Emergency Medicine, Zhongshan Hospital Fudan University, Shanghai, 200032 China; 4 Department of Emergency Medicine, China Medical University First Hospital, Shenyang, 110001 China; 5 Department of Emergency Medicine, The Second Xiangya Hospital of Central South University, Changsha, 410011 China; 6 Department of Emergency Medicine, Peking University Shenzhen Hospital, Shenzhen, 518036 China; 7 Department of Emergency Medicine, Peking University First Hospital, Beijing, 100034 China; 8 Department of Emergency Medicine, The Affiliated Hospital of Innor Mongolia Medical University, Innor Mongolia, 010050 China; 9 Department of Emergency Medicine, China-Japan Friendship Hospital, Beijing, 100029 China

**Keywords:** Cardiac arrest, Cardiopulmonary resuscitation, High-quality cardiopulmonary resuscitation, Cardiopulmonary resuscitation quality control

## Abstract

**Background:**

To investigate current knowledge, attitudes, and practices for CPR quality control among emergency physicians in Chinese tertiary hospitals.

**Methods:**

Anonymous questionnaires were distributed to physicians in 75 tertiary hospitals in China between January and July 2018.

**Results:**

A total of 1405 respondents answered the survey without obvious logical errors. Only 54.4% respondents knew all criteria of high-quality CPR. A total of 91.0% of respondents considered CPR quality monitoring should be used, 72.4% knew the objective method for monitoring, and 63.2% always/often monitored CPR quality during actual resuscitation. The main problems during CPR were related to chest compression: low quality due to fatigue (67.3%), inappropriate depth (57.3%) and rate (54.1%). The use of recommended monitoring methods was reported as follows, ETCO_2_ was 42.7%, audio-visual feedback devices was 10.1%, coronary perfusion pressure was 17.9%, and invasive arterial pressure was 31.1%. A total of 96.3% of respondents considered it necessary to participate in regular CPR retraining, but 21.4% did not receive any retraining. The ideal retraining interval was considered to be 3 to 6 months, but the actual interval was 6 to 12 months. Only 49.7% of respondents reported that feedback devices were always/often used in CPR training.

**Conclusion:**

Chinese emergency physicians were very concerned about CPR quality, but they did not fully understand the high-quality criteria and their impact on prognosis. CPR quality monitoring was not a routine procedure during actual resuscitation. The methods recommended in guidelines were rarely used in practice. Many physicians had not received retraining or received retraining at long intervals. Feedback devices were not commonly used in CPR training.

## Background

The outcomes of Chinese cardiac arrest patients were very poor. In Beijing, the capital of China and where medical technology was well developed, only 1.3% of out-of-hospital cardiac arrest (OHCA) patients were discharged alive, and 1.0% had a favorable neurological outcome (defined as cerebral performance category score of 1 or 2) in 2012 [1]. In 2014, 9.1% of in-hospital cardiac arrest (IHCA) patients were discharged alive and 6.4% had a favourable neurological outcome [2]. The survival and neurological outcomes of OHCA in Beijing did not improve significantly from 2013 to 2017 [3]. The outcomes reported in other cities in China were similar [4–6].

Many factors contributed to these results in China. The current CPR training rate among Chinese residents was less than 1% [7]. Bystander CPR was underutilized, with implementation rates of only 11.4% in Beijing and 4.2% in Shanghai, which were much lower than those in other developed countries [7]. EMS operating mechanism in Chinese city was different. Several cities have implemented dispatcher-assisted CPR, while others would not [8]. The median interval from call receipt to ambulance arrival at the collapse location was usually more than 15 min [3–6]. The number of AEDs per 100,000 residents was lower than that in many developed countries (17.5, 13, and 5 in the developed Chinese cities of Shenzhen, Haikou, and Shanghai, respectively) [9]. Target temperature management for cardiac arrest patients was still in the early stage, and only 7.8% of physicians and 5.7% of nurses had implemented therapeutic hypothermia for cardiac arrest patients [10]. Nevertheless, Chinese emergency physicians have been working hard to improve the prognosis of cardiac arrest patients.

CPR was a lifesaving intervention and the cornerstone of resuscitation. When cardiac arrest took place, blood circulation was completely stopped. CPR could provide 10% to 30% of normal blood flow to the heart and 30% to 40% of normal blood flow to the brain [11]. Early CPR was an important link in the survival chain, regardless of OHCA or IHCA cases [12].

Survival from cardiac arrest depended on early recognition of the arrest event and immediate activation of the emergency response system, but equally critical was that CPR delivered was high-quality. High-quality CPR included ensuring an adequate chest compression rate and depth, allowing full chest recoil between compressions, minimizing interruptions in chest compressions, and avoiding excessive ventilation [13]. There was clear evidence that providing high-quality CPR significantly improves resuscitation outcomes [14–17].

Poor-quality CPR should be considered preventable harm. Monitoring both patient physiologic parameters and provider performance during CPR was essential to optimizing CPR quality [13]. Visual observation was the most basic monitoring method. In 2013, the American Heart Association (AHA) published a consensus statement focused on strategies to improve CPR quality [17]. Patient physiologic parameters during CPR that were considered pertinent for monitoring included invasive hemodynamic data (coronary perfusion pressure > 20 mmHg or arterial diastolic pressure > 25 mmHg) and end-tidal carbon dioxide (ETCO_2_) concentrations > 20 mmHg [17]. Audiovisual feedback devices to measure provider CPR performance were widely accepted [17]. CPR guidelines recommended using feedback devices for real-time optimization of CPR performance [13, 18].

Today, few healthcare organizations consistently applied strategies of systematically monitoring CPR quality even though there was an unacceptable disparity in the quality of resuscitation care and outcomes [13, 18]. As the current knowledge, attitudes, and practices for CPR quality monitoring in China have never been reported, the aim of the present survey was to investigate the awareness and application of CPR quality monitoring among Chinese emergency physicians.

## Methods

### Geographical background

Mainland China was divided into six administrative regions according to geographical distribution, including the eastern region, northern region, northeastern region, southwestern region, southcentral region, and northwestern region. These regions differed in medical development [19].

### Study design

This was a cross-sectional multicenter study. In China, only tertiary, or Level III, hospitals had independent emergency departments and were also teaching hospitals, so these hospitals were identified in an attempt to target providers most likely to care for cardiac arrest patients. Tertiary general hospitals in mainland China were selected by separate random sampling in the six administrative regions. We screened for all 374 tertiary general hospitals, which were then coded randomly by SPSS version 25.0 (IBMCorp, Armonk, New York, USA) and then arranged in ascending order in each administrative region. The top 20% of hospitals were enrolled in the study, with alternative hospitals considered (in ascending order) if any enrolled hospitals were inaccessible or refused to participate. Finally, 75 hospitals were included. We then conducted a questionnaire survey for all emergency physicians of the selected hospitals.

The questionnaire was developed by a senior emergency physician, and examined and discussed three times by an expert team consisting of an epidemiologist and emergency specialists experienced in the management of cardiac arrest patients. The questionnaire included three parts: (1) background data of the respondent; (2) the respondent’s awareness and practices for quality monitoring during actual CPR; and (3) awareness and practices for quality monitoring in CPR training. A total of 30 questions were included in the questionnaire, including 3 blank questions, 20 single choice questions and 7 multiple choice questions.The paper questionnaires were distributed to the directors of emergency departments by mail from January to March 2018. Data collection ended in July 2018.

The study protocol was approved by Peking University Third Hospital Medical Science Research Ethics Committee (Project number: IRB00006761-M2018030, Ethics approval document number: 2018–176-01).

### Statistical methods

The data were analysed by SPSS version 25.0 (IBMCorp, Armonk, New York, USA). Quantitative variables were expressed as the mean (standard deviation) when following a Gaussian distribution, or median (interquartile range 25%-75%) otherwise. Qualitative variables were expressed as frequencies.

## Results

A total of 1489 (93.1%) responses were collected from 1600 questionnaires in 75 hospitals; 1405 responses were analysed and 84 were excluded because of obvious logical errors. The general characteristics of the respondents were described in Table 1.

**Table 1:** Characteristics of respondents

Characteristics	n	(%)
Gender		
Male	812	(57.8)
Female	576	(41.0)
No answer	17	(1.2)
Age (years), mean ± SD^a^	35.28 ± 7.40	
Academic degrees		
Bachelor	532	(37.9)
Master	694	(49.4)
Doctorate	146	(10.4)
No answer	33	(2.3)
Title		
Resident	611	(43.5)
Attending	500	(35.6)
Associate chief physician	209	(14.9)
Chief physician	64	(4.6)
No answer	21	(1.5)
Years of working, median (Q1,Q3)^b^	7(3,13)	
Hospital location		
East region	290	(20.6)
North region	247	(17.6)
Northeast region	209	(14.9)
South-central region	335	(23.8)
Southwest region	199	(14.2)
Northwest region	125	(8.9)
Number of treated cardiac arrest patients per year		
0–10	315	(22.4)
11–30	507	(36.1)
31–50	251	(17.9)
> 50	320	(22.8)
No answer	12	(0.9)
ROSC in treated cardiac arrest patinets		
> 30%	302	(21.5)
21%-30%	303	(21.6)
11%-21%	246	(17.5)
6%-10%	265	(18.9)
< 6%	274	(19.5)
No answer	15	(1.1)
Discharge survival in treated cardiac arrest patinets		
> 30%	137	(9.8)
21%-30%	176	(12.5)
11%-21%	228	(16.2)
6%-10%	311	(22.1)
< 6%	538	(38.3)
No answer	15	(1.1)
Good neurological outcome in treated cardiac arrest patinets		
> 30%	125	(8.9)
21%-30%	153	(10.9)
11%-21%	181	(12.9)
6%-10%	286	(20.4)
< 6%	644	(45.8)
No answer	16	(1.1)

^a^27 respondents did not answer this question

^b^29 respondents did not answer this question

### Knowledge and attitude for high-quality CPR and quality monitoring

Only 54.4% respondents knew all six criteria of high-quality CPR. A total of 60.9% knew all four criteria of high-quality chest compression and 78.2% knew both criteria of avoiding excessive ventilation. A total of 91.0% of respondents considered CPR quality monitoring should be used, and 72.4% knew at least one objective method for CPR quality monitoring. Among the recommended quality monitoring methods, only ETCO_2_ was well known by 71.7% respondents, while others were not.

The knowledge and attitude for CPR quality and quality monitoring were described in Table 2.

**Table 2:** Knowledge and attitude for CPR quality and quality monitoring

Questions and answers	n	(%)
Which of the following criterion are included in high quality CPR? (*n* = 1402)		
ensuring adequate chest compression depth	1309	(93.4)
ensuring adequate chest compression rate	1304	(93.0)
allowing full chest recoil between compressions	1261	(89.9)
minimizing interruptions in chest compressions	956	(68.2)
ensuring adequate rate of ventilation	1209	(86.2)
ensuring adequate volume of ventilation	1158	(82.6)
Should CPR quality be monitored during resuscitation? (*n* = 1387)		
Yes	1278	(92.1)
No	63	(4.5)
I don’t know	46	(3.3)
Should CPR quality be monitored during resuscitation, when use mechanical CPR device? (*n* = 1376)		
Yes	1298	(94.3)
No	40	(2.9)
I don’t know	38	(2.8)
Whether a dedicated person is responsible for quality monitoring? (*n* = 1389)		
Yes	1220	(87.8)
No	91	(6.6)
I don’t know	78	(5.6)
Do you know any objective method or technology of CPR quality monitoring? (*n* = 1400)		
Yes	1014	(72.4)
No	386	(27.6)
How do you learn about monitoring mothed or technique? (*n* = 1014)		
Clinical guideline	857	(84.5)
Academic conference	761	(75.0)
Medical literature	584	(57.6)
Company promotion	112	(11.0)
Others	15	(1.5)
Do you know which of the following quality monitoring methods ? (*n* = 1014)		
Palpation of arterial pulse	831	(82.0)
Observation of the ECG waveforms	759	(74.9)
Observation of the SpO_2_ waveforms	662	(65.3)
Pulse oximetry plethysmographic waveform	484	(47.7)
End-Tidal CO2	727	(71.7)
Coronary perfusion pressure	384	(37.9)
Invasive arterial pressure	566	(55.8)
Audiovisual feedback device	387	(38.2)
Others	8	(0.8)
Do you think CPR quality monitoring could improve CPR quality? (*n* = 1394)		
Yes	1158	(83.1)
No	29	(2.1)
No clear conclusion	120	(8.6)
Don't know relevant research	87	(6.2)
Do you think CPR quality monitoring could improve the return of spontaneous circulation? (*n* = 1391)		
Yes	1094	(78.6)
No	38	(2.7)
No clear conclusion	158	(11.4)
Don't know relevant research	101	(7.3)
Do you think CPR quality monitoring could improve survival? (*n* = 1374)		
Yes	1024	(74.5)
No	36	(2.6)
No clear conclusion	195	(14.2)
Don't know relevant research	119	(8.7)
Do you think CPR quality monitoring could improve the neurologic outcome? (*n* = 1393)		
Yes	993	(71.3)
No	44	(3.2)
No clear conclusion	183	(13.1)
Don’t know relevant research	173	(12.4)

### Practices of CPR quality monitoring

The main problems during CPR were related to chest compression: low quality due to fatigue (67.3%), inappropriate depth (57.3%) and rate (54.1%). A total of 63.2% always and often monitored CPR quality during actual CPR. Methods not recommended by guidelines were usually used for CPR quality monitoring, such as observing ECG waveforms and SpO_2_ waveforms, and pulse oximetry plethysmographic waveforms. In contrast, the methods recommended by guidelines, such as ETCO2, coronary perfusion pressure, invasive arterial pressure and audiovisual feedback devices, were not widely used.

The practices of CPR quality monitoring during actual resuscitation were described in Table 3.

**Table 3:** The practices of CPR quality monitoring during actual resuscitation

Questions and answers	n	(%)
What are the common problems during actual resuscitation? (*n* = 1364)		
Low quality of chest compression due to fatigue	918	(67.3)
Can not ensure appropriate chest compression depth	782	(57.3)
Can not ensure appropriate chest compression rate	738	(54.1)
No full chest recoil between compressions	706	(51.8)
Long interruptions between chest compressions	684	(50.1)
Can not ensure appropriate ventilation rate	667	(48.9)
Can not ensure appropriate ventilation volume	643	(47.1)
Too short ventilation delivery time	430	(31.5)
Insufficient capacity of team leader	219	(16.1)
Poor cooperation between team members	36	(2.6)
Others	24	(1.8)
Do you use mechanical CPR devices during actual resuscitation? (*n* = 1391)		
Always	167	(12.0)
Often	407	(29.3)
Sometimes	335	(24.1)
Rarely	155	(11.1)
Never	327	(23.5)
Which type of mechanical CPR devices do you use? (*n* = 1038)		
Piston device only	770	(74.2)
Load-distributing band device only	81	(7.8)
Both of above	152	(14.6)
Others	35	(3.4)
Do you monitored CPR quality during actual resuscitation? (*n* = 1375)		
Always	485	(35.3)
Often	384	(27.9)
Sometimes	210	(15.3)
Rarely	79	(5.7)
Never	217	(15.8)
Which method do you use for monitoring quality ? (*n* = 1158)		
Palpation of arterial pulse	1010	(87.2)
Observation of the ECG waveforms	994	(85.8)
Observation of the SpO_2_ waveforms	811	(70.0)
Pulse oximetry plethysmographic waveform	274	(23.7)
End-Tidal CO2	495	(42.7)
Coronary perfusion pressure	207	(17.9)
Invasive arterial pressure	360	(31.1)
Audiovisual feedback device	117	(10.1)
Others	6	(0.5)
Does the method you used could achieve the monitoring purposes? (*n* = 1158)		
Completely	75	(6.5)
Most	528	(45.6)
Few	496	(42.8)
Never	50	(4.3)
What is the interval from the start of CPR to start of quality monitoring? (*n* = 1149)		
0-2 min	496	(43.2)
3-6 min	471	(41.0)
7-10 min	100	(8.7)
> 10 min	82	(7.1)
Is there a dedicated person responsible for monitoring during actual resuscitation? (*n* = 1152)		
Always	255	(22.1)
Often	299	(26.0)
Sometimes	289	(25.1)
Rarely	210	(18.2)
Never	99	(8.6)

**Fig. 1:**
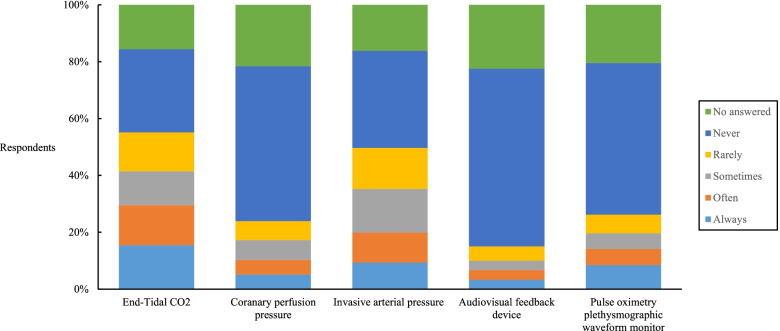
Usage of recommended monitoring technology

**Fig. 2:**
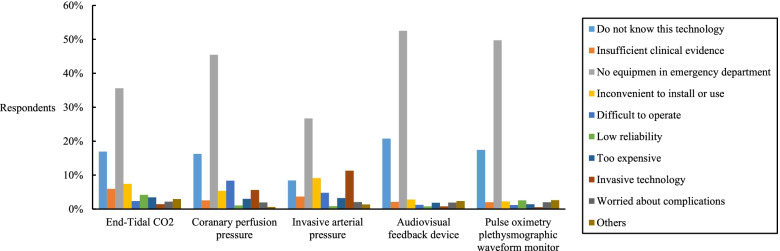
Reasons for not always using recommended monitoring technology

Among the quality monitoring methods recommended by guidelines, ETCO_2_ and invasive arterial pressure were used more often than others. The main reason why these methods were not used was that the emergency department did not have the equipment. The use of recommended monitoring technology and reasons for not always using were described in Fig. 1 and Fig. 2.

### CPR quality monitoring in training

A total of 96.3% of respondents considered it necessary to participate in regular CPR retraining, but 21.4% did not receive any retraining. The ideal retraining interval was considered to be 3 to 6 months, but the actual interval was 6 to 12 months. Only 49.7% respondents reported they always/often used feedback devices in CPR training. Practices and attitudes on quality monitoring in CPR training were described in Table 4.

**Table 4:** Quality monitoring in CPR training

Questions and answers	n	(%)
Training and retraining		
Do you think emergency physicians should participate in regular CPR retraining? (*n* = 1346)		
Yes	1296	(96.3)
No	38	(2.8)
Do not know	12	(0.9)
What is the ideal interval between retraining in your opinion? (*n* = 1294)		
3 months	443	(34.2)
6 months	500	(38.6)
12 months	272	(21.0)
24 months	71	(5.5)
Other interval	8	(0.6)
Have you attend CPR training or retraining as a trainee? (*n* = 1344)		
Yes	1057	(78.6)
No	287	(21.4)
What is the ideal interval between your retraining in actual? (*n* = 1043)		
3 months	283	(27.1)
6 months	332	(31.8)
12 months	333	(31.9)
24 months	79	(7.6)
Other interval	16	(1.5)
CPR feedback devices		
Do you think using CPR feedback devices could improve CPR performance in training? (*n* = 1343)		
Yes	1210	(90.1)
No	45	(3.4)
Do not know	88	(6.6)
Did you used CPR feedback devices in your actual training or retraining? (*n* = 1052)		
Always	222	(21.0)
Often	303	(28.7)
Sometimes	178	(16.8)
Rarely	84	(7.9)
Never	265	(25.1)
Which part of training dos CPR feedback devices usually be used? (*n* = 787)		
Skill training	543	(69.0)
Practice	581	(73.8)
Test	476	(60.5)

## Discussion

### Knowledge and attitudes for CPR quality monitoring

We found a good situation in which 92.1% of respondents considered CPR quality monitoring to be needed during resuscitation. Mechanical compression devices were designed for some special situations and were considered to reduce the physical burden of emergency physicians [20]. Because emergency departments in China were often crowded, the use of mechanical devices during resuscitation was common. Even in this situation, 94.3% of respondents considered CPR quality to need to be monitored. This showed that emergency physicians were concerned about CPR quality.

High-quality CPR was an important link between survival chains, and it may be more important than other links [12]. Unfortunately, only 54.4% of respondents in this survey knew all six criteria of high-quality CPR and 60.9% knew all four criteria of high-quality chest compression. Most respondents were concerned about chest compression depth, rate and chest recoil between compressions. However, it was concluded that emergency physicians in tertiary general hospitals did not pay enough attention to minimizing compression interruptions. Minimizing compression interruption was also an important criterion of high-quality CPR emphasized in guidelines [13, 18]. Continuous chest compressions could maintain adequate coronary perfusion pressure, and increase the likelihood of ROSC [21]. Chest compression fraction over 80% was recommended to ensure that compressions were continued with as few interruptions as possible during CPR. However, in this survey, only 68.0% of respondents knew this criterion. This may become a problem for improving CPR quality and it was important to strengthen the education of emergency phycisians to minimize compression interruption.

Visual observation was the most basic and widely used monitoring method, but objective parameters were recommended for accurate monitoring [17]. We found that 72.4% of respondents knew objective monitoring methods. Clinical guidelines and academic conferences were the main ways emergency phycisians learned about their technology. However, in this survey, Chinese emergency physicians did not know some recommended techniques, especially audiovisual feedback devices.

It was important to find that many respondents had some misunderstandings about CPR quality control. For example, 71.3% of Chinese emergency physicians considered that using CPR quality monitoring devices could improve cardiac arrest patients’ outcomes. While current studies have not demonstrated significant improvement in outcomes related to CPR quality monitoring during resuscitation [13, 17, 22].

Although Chinese emergency physicians were concerned about CPR quality, they lacked an understanding of high-quality CPR criteria, new objective monitoring methods and research results.

### Practices of CPR quality monitoring

Chest compression quality was a main problem during actual resuscitation in China. Low quality due to fatigue, inappropriate compression depth, and inappropriate compression rate were the top three problems reported by respondents. In contrast, personal ability and team cooperation were not issues. Because of this, quality control should be placed on a vital position in China. However, CPR quality monitoring was not a routine procedure in Chinese emergency department. Only 63.2% of respondents reported that they always/often monitored CPR quality during actual resuscitation. This showed that Chinese emergency physicians did not comply with the guidelines for CPR quality monitoring.

Accurate measurement of CPR quality was a precondition for high quality CPR. Objective parameters were better than visual observation [13]. Our results showed that recommended methods were rarely used in Chinese emergency departments. ETCO_2_ was the most widely used among these methods. The opinions and clinical experience of experts strongly supported using ETCO_2_ to optimize chest compression quality during resuscitation [13, 17]. Endotracheal intubation was not difficult for Chinese emergency physicians, because they had rich clinical experience and visualization devices were widely used. Previous studies showed that advanced airway could be placed in the first few minutes during resuscitation in Chinese emergency departments [23]. In this situation, ETCO2 data was easier to obtain. This could explain why ETCO_2_ was widely used in China. However, Using an audio-visual feedback device to monitor CPR quality was another recommended method by guidelines [13, 18]. It was a noninvasive technology for real-time monitoring, recording, and feedback about CPR performance [24, 25]. We found that less than 20% of respondents had used this equipment. Pulse oximetry was widely used, and its waveform could reflect peripheral tissue perfusion. Some research found that the appearance of pulse oximetry plethysmographic waveforms was related to CPR quality [26, 27]. Pulse oximetry plethysmographic waveforms, as a monitoring technology, were recommended for CPR quality monitoring by Chinese expert consensus in 2018. However, its usage rate was still lower than that of ETCO_2_ and invasive arterial pressure. The survey reported that few emergency departments had such equipment, and many emergency physicians did not know this technology.

Unrecommended methods were widely used for quality monitoring in Chinese emergency department and was the biggest problem. Palpation of the arterial pulse, observation of the ECG waveform, and observation of the SpO_2_ waveform were the top three most widely used methods. Palpation of arterial pulse was the most widely used method to evaluate chest compression quality, but it has been shown to be unreliable and cannot be used for continuous monitoring during actual resuscitation [28, 29]. Therefore, the guidelines did not recommend this method for CPR quality monitoring [17]. Regular ECG waveforms accompanying chest compression can be observed in some patients, but the shape of the waveform had no clear relationship with the quality of chest compressions [30]. The ECG waveform was widely used for quality monitoring, reflecting the misunderstanding of its meaning in Chinese emergency physicians.

Continuing education for emergency physicians was crucial to increase the use of recommended methods [31]. Although CPR quality monitoring had been recommended by CPR guidelines, fewer specific consensus protocols existed that provided detail on how to better implement the monitoring. The development and publication of standardized monitoring protocols would likely help physicians better implement CPR quality monitoring in China.

### CPR quality monitoring in training

Basic life support and advanced cardiac life support techniques were the core skills of resuscitation [32]. The CPR training course was a key part of Chinese resident standardized training program. In most tertiary hospitals, all emergency physicians needed to attend basic life support courses and many of them also needed to attend advanced cardiac life support courses. CPR training courses in many hospitals were certified by the American Heart Association [33].

CPR training was not a one-time training. Retraining was recommended by AHA guidelines, because skills and knowledge may decay within 3 to 12 months after initial training [32]. The concept of retraining was widely accepted in China, where 96.3% of respondents believed it was necessary to retrain after initial training. Responses showed that CPR retraining received great attention, and that a “frequent” retraining concept was more acceptable. A total of 72.8% of respondents considered the ideal interval between trainings to be 3 to 6 months, although there was no clear recommendation on the optimal time interval [32]. Unfortunately, the survey results revealed a large gap between attitudes and practice, as 21.4% of emergency physicians did not receive any retraining after initial training. Among those who did, the actual retraining interval was 6 to 12 months, significantly longer than desired. This showed that most hospitals did not have standard retraining systems. Retraining may be difficult to implement in some hospitals. Short-term frequent retraining may be a solution in China. Because it would not increase the cost, if the total training time was fixed. Physicians were more likely to take part in a short training course after busy work. And frequent retraining was helpful to consolidate skills.

According to AHA’s CPR guidelines, feedback devices should be used in CPR training [32]. This attitude was supported by 90.1% of respondents, who believed that CPR feedback devices can improve performance during training. However, attitudes and practice was so different. The typical training course in Chinese hospitals includes two parts: theory training and skills training. While the structure of the training course was reasonable, only 49.7% of respondents replied that CPR feedback devices were always/often used in training, and 25.1% replied that they never used feedback devices. In developing countries such as China, there were many hospitals that have no ability to purchase these devices. This may become an important barrier to improving CPR quality.

## Conclusions

In this survey we found that Chinese physicians in tertiary hospitals were very concerned about CPR quality, but they did not fully understand the high-quality criteria and their impact on prognosis. Most emergency physicians considered it necessary to monitor CPR quality, but quality monitoring was not a routine procedure during actual resuscitation. Recommended monitoring methods, such as audio-visual feedback devices, were rarely used in practice. However, many physicians used unrecommended methods. Although retraining was considered important, many physicians had not received retraining or received retraining at long intervals. Feedback devices were not widely used in CPR training.

In the future, Chinese emergency physicians should receive systematic continuing education on CPR quality control. A standard operation procedure should be established to guide CPR quality monitoring during actual resuscitation, including hemodynamic parameters, ETCO_2_ and audio-visual feedback devices. Retraining plans and feedback devices should also be an integral part of CPR training.

## Limitations

Emergency departments of tertiary hospitals were targeted for this survey. Physicians in these hospitals were likely to have the most experience caring for cardiac arrest patients. At the same time, these hospitals had the most advanced equipment. The overrepresentation of these hospitals may have subjected the survey to bias. In other words, it was likely that the CPR quality monitoring responses reflected in our survey demonstrated an optimistic perspective. The true proportions of knowledge, attitudes, and practices of monitoring across all Chinese hospitals may be lower than those reported in this survey. Additionally, although the majority of published examples of quantifying qualitative data used dichotomous variables for simplicity, such conversion may result in overestimation or underestimation due to identical grading of responses such as “usually” and “always” and “sometimes” and “never.”

## Data Availability

The datasets used and/or analysed during the current study are available from the corresponding author on reasonable request.

## Abbreviations

OHCA: Out-of-hospital cardiac arrest
IHCA: In-hospital cardiac arrest
CPR: Cardiopulmonary resuscitation
AHA: American Heart Association
ETCO_2_: End-tidal carbon dioxide
